# Activation of AcvR1-Mediated Signaling Results in Semilunar Valve Defects

**DOI:** 10.3390/jcdd9080272

**Published:** 2022-08-16

**Authors:** Shabber Syed, Sudha Rajderkar, Jeffrey M. Mann, Travis Hawkins, Bingrou Wu, Bin Zhou, Yukiko Sugi, Yuji Mishina, Vesa Kaartinen

**Affiliations:** 1 Department of Biologic and Materials Sciences, University of Michigan School of Dentistry, Ann Arbor, MI 48109, USA; 2 Department of Regenerative Medicine and Cell Biology, Medical University of South Carolina, Charleston, SC 29425, USA; 3 Department of Genetics, Albert Einstein College of Medicine, Bronx, NY 10461, USA

**Keywords:** valve defects, TGF-beta superfamily signaling, calcification

## Abstract

Calcific aortic valve disease (CAVD) is a common cardiac defect, particularly in the aging population. While several risk factors, such as bi-leaflet valve structure and old age, have been identified in CAVD pathogenesis, molecular mechanisms resulting in this condition are still under active investigation. Bone morphogenetic protein signaling via the activin type I receptor (AcvRI) plays an important role during physiological and pathological processes involving calcification, e.g., bone formation and heterotopic ossification. In addition, AcvRI is required for normal cardiac valve development, yet its role in aortic valve disease, if any, is currently unknown. Here, we induced the expression of constitutively active AcvRI in developing mouse embryos in the endocardium and in cells at the valve leaflet–wall junction that are not of endocardium origin using the *Nfac1Cre* transgene. The mutant mice were born alive, but showed thickened aortic and pulmonary valve leaflets during the early postnatal period. Adult mutant mice developed aortic stenosis with high frequency, sclerotic aortic valves, and displayed Alcian Blue-positive hypertrophic chondrocyte-like cells at the leaflet–wall junction. Calcification was only seen with low penetrance. In addition, we observed that the expression levels of gene sets associated with inflammation-related cytokine signaling, smooth muscle cell contraction, and cGMP signaling were altered in the mutants when compared with those of the controls. This work shows that, in a mouse model, such continuous AcvRI activity in the *Nfatc1Cre* recombination domain results in pathological changes in the aortic valve structure and function.

## 1. Introduction

Cardiac valve defects are among the most common congenital birth defects in humans [1]. They can be isolated [2], or they can occur in conjunction with other congenital cardiac defects or other known syndromes affecting multiple organs [3,4,5,6]. Cardiac valves are needed for coordinated unidirectional blood flow. Valve development starts with the formation of endocardial cushions in the outflow tract (OFT) and in the atrio-ventricular junction (AVJ) of the embryonic heart. Hyaluronic-rich extracellular matrix (cardiac jelly) is deposited between the endocardial and myocardial cell layers, followed by mesenchymal cell invasion into the acellular cushions. In the AVJ and proximal OFT, these mesenchymal cells are formed from the endocardium via endocardial-to-mesenchymal transformation (EndMT), while the distal OFT cushions are populated by mesenchymal cells that have their origin in the cardiac neural crest [7]. In addition, the second heart field makes an important contribution to aortic valve development [8,9]. Subsequently, the OFT endocardial cushions undergo complex steps of remodeling and maturation, resulting in the formation of functional cardiac valve leaflets, which are composed of three stratified layers of extracellular matrix interspersed with valve interstitial cells (VICs) [10]. These steps are controlled by morphogenetic signaling pathways and their target transcription factors. For instance, bone morphogenetic proteins (BMPs) induce the key chondrogenic factor Sox9, which is required for cardiac valve precursor cell expansion and ECM organization [11,12].

Bone morphogenetic proteins (BMPs) belong to the TGF-β superfamily of growth factors, which, together with other morphogens, regulate both embryogenesis and postnatal tissue growth and homeostasis [13,14,15]. They were originally discovered as “bone-forming” factors [16], but were subsequently shown to play critical roles in practically all cell types and organ systems during embryogenesis, tissue homeostasis, regeneration, and aging [17,18]. In addition, it has been shown that uncontrolled or defective BMP signaling in humans can result in pathological conditions, e.g., congenital birth defects and malignant growth [19,20,21].

Activin receptor I (AcvRI, Alk2) is one of the BMP type I receptors [13,22]. In humans, loss of function mutations in the *ACVR1* gene result in congenital cardiac defects affecting the valve and septum formation [23,24], while gain of function mutations lead to the rare but fatal fibrodysplasia ossificans progressiva (FOP) disease, characterized by aberrant heterotopic ossification [25]. Similarly, in transgenic animal models, constitutive activation of AcvRI has been shown to result in ectopic bone formation [26,27], whereas loss of function mutations result in different cardiac defects depending on the cell type harboring the mutated receptor [28,29]. Using an accurate genetic mouse model for FOP (*Acvr1^R206H^*), Lees-Shepard et al. were able to show that fibro/adipogenic progenitor cells are a major contributor to heterotopic osteogenic changes in FOP disease [30]. Our previous studies showed that, during mouse embryogenesis, the *Acvr1* gene is exceptionally strongly expressed in endocardial cells, and that cell type-specific deletion of *Acvr1* in endothelial cells and in the cushion mesenchyme results in defective endocardial cushion formation and in valve defects, respectively [31,32]. However, the effect of enhanced AcvRI-mediated signaling on cardiac valve development is currently unknown. Herein, we show that *Acvr1* is expressed in several cardiac cell types in postnatal mice, and that activation of AcvRI in the *Nfatc1Cre* recombination domain (endocardium and its derivatives, as well as the leaflet–wall junction) results in the formation of thickened semilunar valves, chondrogenic changes in the valve leaflet–wall junction, and functional aortic valve defects.

## 2. Materials and Methods

Mice: Transgenic mice carrying the Cre-activatable constitutively active *Acvr1* transgene (caAcvr1) and knock-in mice carrying the ires-Cre-PA cassette in the Nfatc1 locus (*Nfatc1Cre*) have previously been described [33,34]. Mutant (caAcvr1(+):*Nfatc1Cre* (+)) and control (either caAcvr1(−):*Nfatc1Cre* (+), caAcvr1(+):*Nfatc1Cre* (−), or caAcvr1(−):*Nfatc1Cre* (−)) embryos/mice were obtained by crossing *caAcvr1* mice with *Nfatc1Cre* mice. *Gt(ROSA)26Sor^tm4(ACTB-tdTomato,-EGFP)Luo^*/J was obtained from the Jackson Laboratory (stock #7576). All of the experiments involving the use of mice were carried out according to federal and institutional guidelines (protocol number PRO00007157).

Histology, immunohistochemistry, and *X-gal* staining: For paraffin embedding, tissues were fixed in 4% p-formaldehyde (pFA) overnight at 4 °C and processed through a graded series of ethanol for paraffin embedding. Embedded tissues were sectioned (7 μm). Hematoxylin and Eosin staining was performed according to standard procedures. For frozen sectioning, fixed tissues (4% pFA overnight) were processed to 30% sucrose in PBS, embedded in OCT, and cryosectioned (10 μm). For *lacZ*-staining, tissues were fixed at 4% pFA for 15 min, washed in a detergent wash solution, processed through 10% and 30% sucrose in PBS solutions (overnight), and embedded either in the Tissue-Tek^®^ OCT compound or 7% gelatin/15% sucrose [29]. After cryosectioning (10 μm), the sections were stained in the X-gal staining solution described in [35]. After overnight staining, the sections were postfixed, dehydrated, mounted on glass slides, and photographed. For immunohistochemistry, antigens were retrieved using the acidic citrate method. The following antibodies were used: Sox9 (Santa Cruz sc20095), Aggrecan (Chemicon AB1031), Wnt7a (LSBio LS-C335567), and Opg (Sigma O1139). Primary antibodies were detected using Alexafluor-488-labeled secondary antibodies (Life Sciences Technology), and the sections were mounted with Vectashield (with DAPI) (Vector Labs Inc., Burlingame, CA, USA). Masson’s trichrome staining was performed using a Trichrome Kit (Sigma-Aldrich, St. Louis, MO, USA) according to the manufacturer’s instructions. For Alcian Blue, sections were stained for 15 min in an Alcian Blue staining solution (0.1%/0.5% acetic acid, pH 3.1), followed by counterstaining with nuclear fast red (Vectastain). Alizarin Red staining was performed as described in [35].

Echocardiography, blood pressure, and heart rate measurements (these analyses were performed at Frankel Cardiovascular Center Physiology and Phenotyping Core): Anesthesia was induced in an enclosed container filled with 4% isoflurane. Subsequently, the mice were placed on a warming pad to maintain body temperature. Then, 1–1.5% isoflurane was supplied via a nose cone. The hair was removed from the upper abdominal and thoracic area with depilatory cream. ECG was monitored via non-invasive resting ECG electrodes. Transthoracic echocardiography was performed in the supine or left lateral position. Two-dimensional, M-mode, Doppler, and tissue Doppler echocardiographic images were recorded using a Visual Sonics’ Vevo 770 high-resolution in vivo micro-imaging system. The LV ejection fraction was measured from the two-dimensional long axis view. The systolic and diastolic dimensions and wall thickness were measured using the M-mode in the parasternal short axis view at the level of the papillary muscles. Fractional shortening and ejection fraction were also calculated from the M-mode parasternal short axis view. Diastolic function was assessed by conventional pulsed-wave spectral Doppler analysis of mitral valve inflow patterns (early (E) and late (A) filling waves). Doppler tissue imaging (DTI) was used to measure the early (Ea) diastolic tissue velocities of the septal and lateral annuluses of the mitral valve in the apical four-chamber view. Echo was performed on four-month-old *caAcvr1:Nfatc1Cre* mutants, along with male and female control animals of the same ages. Blood pressure and heart rate were measured using the IITC Life Science tail cuff plethysmography blood pressure system (Frankel Cardiovascular Center Physiology and Phenotyping Core).

RNA-Seq: Aortic valve tissues were collected in 100–200 μL of commercially available (Qiagen, Hilden, Germany) RLT buffer. Total RNAs were isolated using a Qiagen RNeasy Mini Kit, Cat. No. 74104. Sequencing libraries were prepared by the University of Michigan DNA Sequencing Core and reads were generated on Illumina HiSeq4000. The read files were downloaded and concatenated into a single.fastq file for each sample. The quality of the raw read data for each sample was checked using FastQC (version 0.10.0) to identify the features of the data that may indicate quality problems (e.g., low-quality scores, over-represented sequences, and inappropriate GC content). The Tuxedo Suite software package was used for alignment, differential expression analysis, and post-analysis diagnostics. Reads were aligned to mm^10^ (mus musculus assembly July 2011) using Bowtie2 (version 2.1.0). Cufflinks/CuffDiff (version 2.1.1) was used for expression quantitation and differential expression analysis, using reference genome sequence reference transcriptome annotation (GEO Submission GSE122106). Impacted signaling pathways and Gene Ontology terms were identified using iPathwaysGuide (Advaita).

Statistical assays: Three or more independent samples were analyzed in each assay (details given in figure texts). The averages, standard errors, and probabilities (non-parametric Mann–Whitney test) were calculated, and *p*-values of less than 0.05 were marked as significant.

## 3. Results

### 3.1. Acvr1 Is Expressed in Adult Semilunar Valves

**Figure 1:**
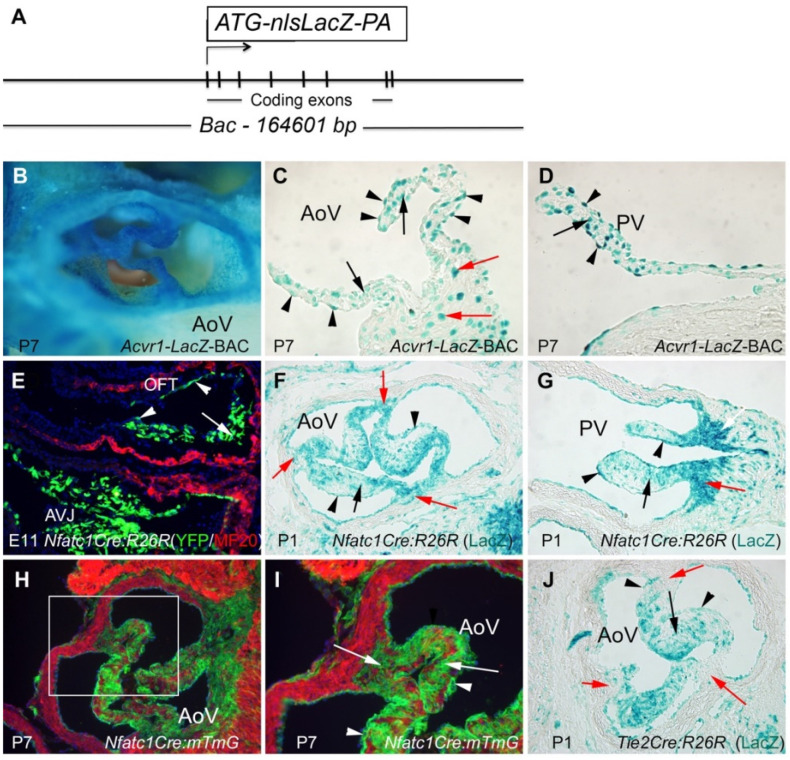
Endocardial cells covering postnatal semilunar valves expressed *Acvr1*. (**A**) Schematic presentation of the 164 kb *Acvr1-lacZ* reporter construct (nls, nuclear localization signal). (**B**) A whole mount view of the X-gal-stained aortic valve (P7, postnatal day 7). (**C**,**D**) Sections depicting X-gal-stained aortic valve (AoV) land pulmonary valve (PV) leaflets at P7. Black arrowheads point to endocardial cells and black arrows point to interstitial cells (VIC). Red arrows (in **C**) point to cells in the leaflet–wall junction region. (**E**) A section showing the OFT and AVJ of *Nfatc1Cre:R26* (YFP) reporter mice (E11). Green cells illustrate recombined cells. White arrowheads in the OFT region point to endocardial cells and white arrows point to proximal OFT cushion cells. MF20 was used to visualize myocardial cells (red). (**F**,**G**) Sections depicting views of the P1 aortic (AoV) and pulmonary (PV) valves of *Nfatc1Cre:R26* reporter mice. Black arrowheads point to endocardial cells, black arrows point to VICs, and red arrows point to positively stained cells in the leaflet–wall junction region. X-gal staining. (**H**) A section showing the P7 aortic valve (AoV) of *Nfatc1Cre:mTmG* reporter mice. Recombined cells, green; unrecombined cells, red. (**I**) High-power images of the field boxed in (**H**). (**J**) A section showing a view of the P1 aortic (AoV) valve of the *Tie2Cre:R26 (LacZ)* reporter mouse. Black arrowheads point to endocardial cells, black arrows point to VICs, and red arrows point to the leaflet–wall junction region, which shows only a few positively stained cells. X-gal staining.

**Figure 2:**
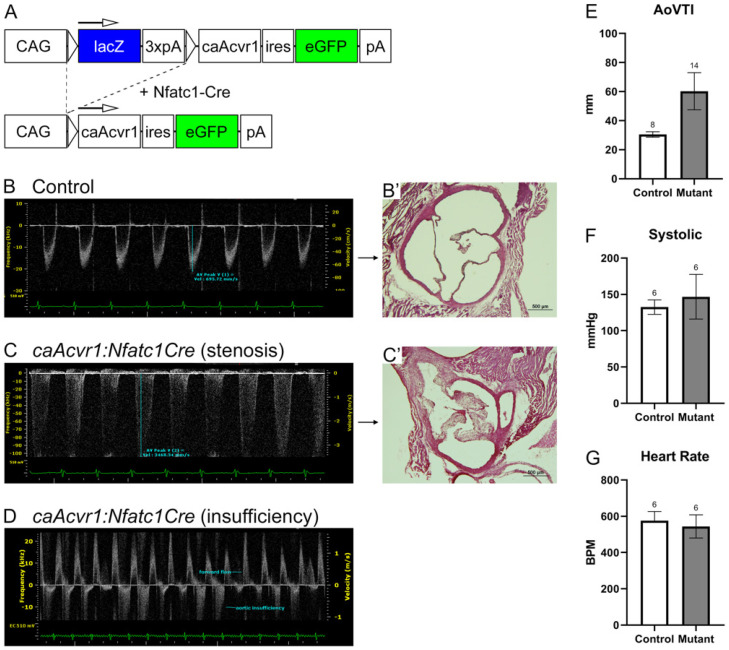
*caAcvr1:Nfatc1Cre* mutants suffered from functional aortic valve defects. (**A**) Schematic presentation of the Cre-activatable *caAcvr1* transgene design. (**B**,**C**) Echocardiography of a control (**B**) and *caAcvr1:Nfatc1Cre* mutants (four months old) with aortic valve insufficiency (**C**) and aortic stenosis (**D**) ((**B**’,**C**’) show the aortic valve histology of the corresponding samples shown in (**B**,**C**)). A bar graph (**E**) depicts a difference in the aortic velocity time integral (AoVTI, mm) between the controls and mutants. No differences in blood pressure ((**F**), mmHg) or heart rate ((**G**), BPM) were detected between the controls and mutants (white columns, controls; gray columns, mutants; error bars, SEM; * *p* < 0.05; *n* = 8 controls and 16 mutants).

To identify cell types expressing *Acvr1* in postnatal mice, we used the *Acvr1-nlsLacZ-BAC* reporter mouse line (Figure 1A), which has been shown to faithfully recapitulate endogenous gene expression [29]. Most of the endocardial cells covering both the aortic and pulmonary valve leaflets, as well as a subset of valvular interstitial cells (VICs) and valve wall cells, were stained positive for reporter activity (Figure 1B–D). A recently included *Nfatc1Cre* mouse line [34] induced robust recombination in endocardial cells, including those covering the OFT and AVJ cushions at E11 (Figure 1E) and the aortic and pulmonary valves at P1–P7 (Figure 1F–I). In addition, *Nfatc1Cre*-induced recombination was seen in the endocardium-derived mesenchymal cells of the OFT and AVJ cushions, in VICs, and in cells in the leaflet–wall junction region (Figure 1E–I), but not in the myocardium or smooth muscle. The number of positively stained VICs, particularly of the cells in the leaflet–wall junction region, appeared to be higher in samples harvested from *Nfatc1Cre* mice than in those harvested from *Tie2Cre* mice (Figure 1J), suggesting that some of the positively stained cells are not of endocardial origin in *Nfatc1Cre* mice. To examine the effect of constitutive activation of AcvRI on cardiac valve development (Figure 2A), the *Nfatc1Cre* knock-in mice were crossed with mice carrying the Cre-inducible constitutively active (Q207D) transgene [33].

### 3.2. caAcvr1:Nfatc1Cre Mutants Are Viable, but Show Functional Defects in the Heart

Genotypically mutant mice were born alive and appeared grossly normal until approximately 10 days of age. Subsequently, they became distinguishable from the control littermates by their smaller size and shorter tails. In addition, they showed accumulation of ectopic cartilage and bone at their distal joints, particularly in their hind limbs [26]. Despite these pronounced external phenotypes, many of the mutant mice survived to adulthood. Owing to worsening limb defects, the mutant mice gradually became poorly ambulatory and had to be euthanized at four months of age. The cardiac function of adult *caAcvr1:Nfatc1Cre* mice was analyzed by echocardiography (Figure 2). In addition, heart rate and blood pressure measurements were recorded (Figure 2). The most common finding was aortic valve stenosis, present in approximately 50% of the examined mutants (8/16) (Figure 2E). Other findings included aortic insufficiency (4/16; Figure 2F) and elevated mitral inflow velocity (2/16) (data not shown). *CaAcvr1* mice without the *Nfatc1Cre* transgene or *Nfatc1Cre* mice with the *caAcvr1* transgene did not show any observable phenotypes and were used as controls. Taken together, the enhancement in AcvRI-mediated signaling in valve precursor cells resulted in partially penetrant defects in aortic valve functioning.

### 3.3. caAcvr1:Nfatc1Cre Mutants Show Thickened Valve Leaflets and Wall Defects

**Figure 3:**
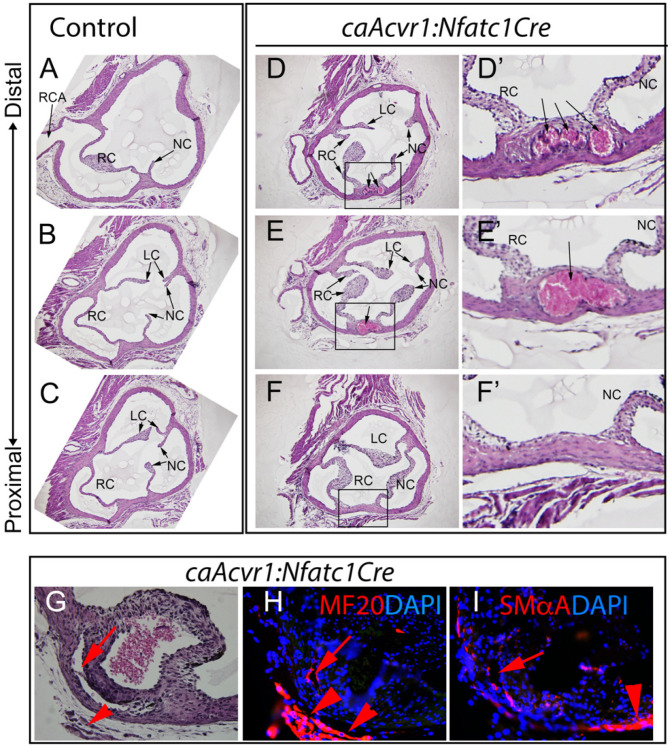
Enhanced AcvRI signaling led to thickened aortic valve leaflets and aortic wall defects. Histological sections of the control (**A**–**C**) and mutant (**D**–**F**) samples (four months old) depicting the aortic valve leaflets (proximal-to-distal) in a transverse orientation. LC, left coronary; RC, right coronary; NC, non-coronary. (**D’**–**F’**) High-power images depicting the aortic wall lesions (boxed areas in (**D**–**F**)). Black arrows in (**D**,**E**) (boxed area) and (**D’**,**E’**) point to the filled sinuses seen in the mutants. (**G**–**I**) Aortic wall defects in the *caAcvr1:Nfatc1Cre* mutants. (**G**) H&E staining. (**H**) MF20 immunostaining. (**I**) SmαA immunostaining. Red arrows in (**G**–**I**) point to aberrant sinuses; red arrowheads in (**G**–**I**) point to adjacent myocardium. (**H**,**I**) Counterstaining with DAPI (blue).

Previous studies have shown that AcvRI is required for endocardial-to-mesenchymal transformation (EndMT), a critical step in normal endocardial cushion development in both OFT and AVJ [36]. As the *Nfatc1Cre* driver becomes active by this time, we examined the OFT cushions at E11.5 to determine if the postnatal valve function might be contributed to by abnormalities in this early event. Both the control and mutant cushions appeared grossly similar in size and shape, suggesting that constitutive activation did not result in excessive EndMT during the development of cardiac valve primordia (Figure S1A,B, Supplementary Materials). Furthermore, other morphological processes sensitive to cushion abnormalities (heart looping, OFT rotation, and AV and OFT septation) occurred successfully in the AcvRI mutants (data not shown), suggesting that OFT and AV cushion development was not drastically affected during gestation. Next, we examined early neonatal (P3) aortic valve morphology by serial sectioning. All of the hearts had three aortic valve leaflets, but those in the mutants were all noticeably thicker than those in the littermate controls (Figure S1C,D). Consistent with the histological findings at P3, the aortic valve leaflets in the adult (four-month-old) mutants were also significantly thicker than those of the littermate controls in both the transverse (Figure 3) and sagittal (Figure S2) orientations relative to the aortic trunk. In addition, aberrant blood-filled sinuses were found in both the interleaflet triangle extending into the apex of the commissure of adjacent right and non-coronary leaflets (Figure 3E,F,H,I), and proximal and lateral to a non-coronary leaflet sinus wall (Figure 3E,F,E’,F’). Similar thick leaflets and wall defects, as well as occasional bi-leaflet valve structures, could be seen in mutant pulmonary valves (data not shown). To examine whether the sinus formed at the border between the cardiac fibrous skeleton and muscle layers, immuno-fluorescence was used to detect smooth (anti-SmαA) and cardiac muscle (MF20) (Figure 3H,I). This demonstrated that the aberrant blood-filled sinuses were formed within the fibrous tissue, and not in the interphase between the fibrous tissue and differentiated muscle.

### 3.4. Hypertrophic Cartilage-like Cells and Calcification in caAcvr1:Nfatc1Cre Mutant Valves

**Figure 4:**
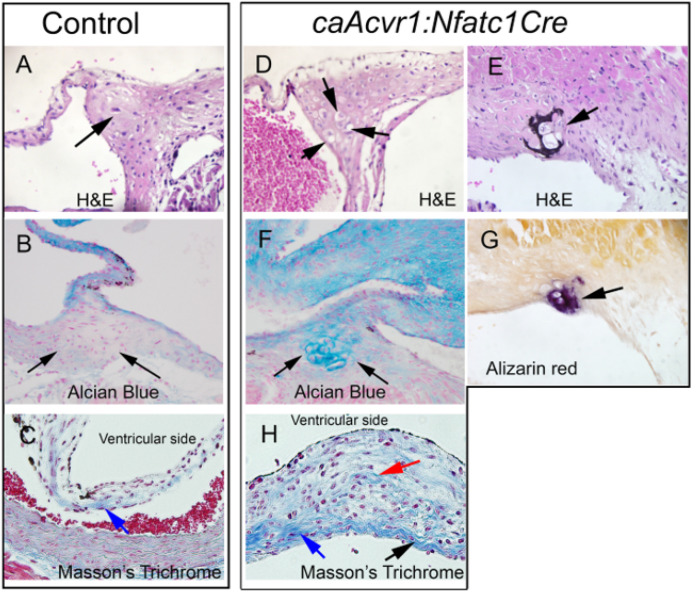
Chondrogenic changes and sporadic calcification in the aortic valve leaflet–wall junction region in the *Acvr1:Nfatc1Cre* mutants. (**A**–**C**) Aortic leaflet and the leaflet–wall junction in the controls. (**D**–**H**) Aortic leaflet and the leaflet–wall junction in the *caAcvr1:Nfatc1Cre* mutants. (**A**,**D**,**E**) H&E staining. (**B**,**F**) Alcian Blue staining. (**C**,**H**) Masson’s trichrome. (**G**) Alizarin Red staining. Arrows in (**A**,**D**) point to the leaflet–wall junction, where hypertrophic chondrocyte-appearing cells can be seen in the mutants (*n* = 6). Arrows in (**B**,**F**) point to the leaflet–wall junction, which was stained positive for Alcian Blue in the mutants (*n* = 6). Arrows in (**C**,**H**) point to the fibrillar collagen-positive fibrosa layer, which appears much thicker in the mutants (*n* = 6). Arrows in (**E**,**G**) indicate calcific lesions in the mutant sample.

Further histological examination revealed that, in the adult mutants, hypertrophic chondrocyte-like cells could be found in the distinctive fibrous cord at the aortic valve leaflet–wall junctions (compare Figure 4A,D), a region recombined by *Nfatc1Cre* (Figure 1F,H,I). These cells were surrounded by the proteoglycan-rich matrix, as indicated by Alcian Blue staining (Figure 4F). Moreover, a subset of the mutants (1/6 *caAcvra:Nfatc1Cre*, 0/6 controls) displayed Alizarin Red-positive calcium deposits (Figure 4E,G). Although no overt chondrocytic or mineralized areas were present in the mutant leaflets themselves, fibrillar collagen, usually restricted to the “fibrosa” layer predominantly at the aortic face of the leaflet [37] (blue stain in the control, Figure 4C), could be detected in a greater amount and more widely distributed throughout thickened mutant leaflets (Figure 4H), suggesting that, in addition to leaflet thickening, the matrix architecture was altered in the aortic valve leaflets of the *caAcvr1:Nfatc1Cre* mutants.

### 3.5. Enhanced AcvRI Signaling Resulted in Changes in the Gene Sets Associated with Cytokine Signaling, Vascular Smooth Muscle Contraction, and cGMP Signaling

**Figure 5:**
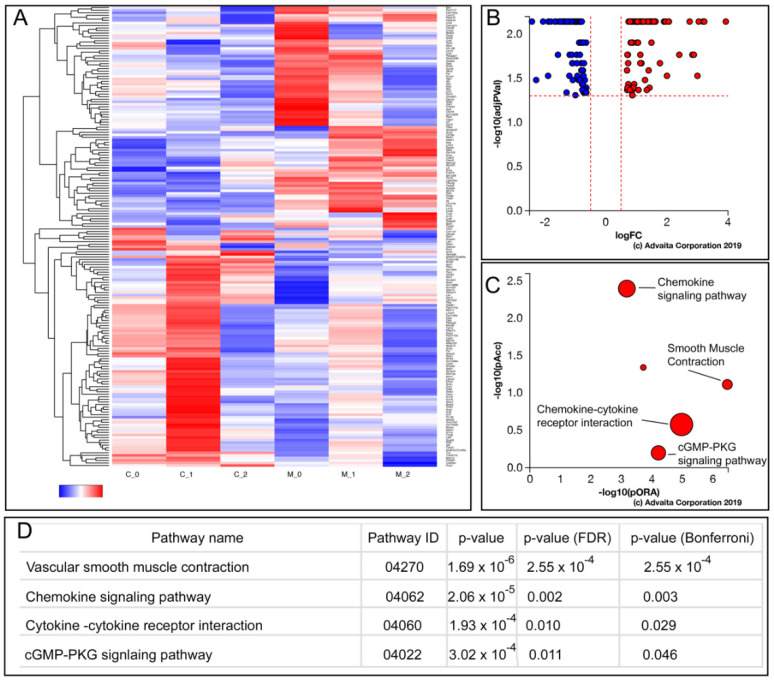
Gene sets associated with smooth muscle cell contraction, chemokine signaling, and cGMP signaling were affected in the *caAcvr1:Nfatc1Cre* mutants. (**A**) Heat maps of the upregulated and downregulated genes in mutant aortic valve tissues. (**B**) Volcano plot showing all 181 significantly differentially expressed genes in terms of their measured expression change (x-axis) and the significance of the change (y-axis). (**C**) Pathway perturbation vs. over-representation. The top five pathways plotted in terms of the total pathway accumulation (y-axis) and over-representation (x-axis). (**D**) Top pathways and their associated *p*-values.

Next, we harvested aortic valve tissues from four-month-old control and mutant mice and subjected them to genome-wide RNA-Seq transcriptome analysis. Of the 181 differentially expressed genes (*p* < 0.05), 93 were up-regulated (fold change ≥ 0.17) and 88 were down-regulated (fold change ≤ 1.5). Pathway analysis (iPathway, Advaita) showed that the genes associated with cytokine signaling and vascular smooth muscle contraction were among the most affected in the *caAcvr1* mutant aortic valve tissues (Figure 5). The BMP synexpression genes (*Smad6, Bambi, Bmpr2*, and *Msx1*) were not altered in the mutant aortic valve tissues, suggesting that BMP signaling was not any more elevated in the adult valve leaflets of the *Acvr1:Nfatc1Cre* mutants. Similarly, the synexpression genes of canonical Wnt signaling (*Axn2* and *Fzd1*), Fgf signaling (*Spry2* and *Erm*), or Shh signaling (*Ptch1* and *Sall1*) were not affected in the mutant valve tissues compared with those of the controls.

**Figure 6:**
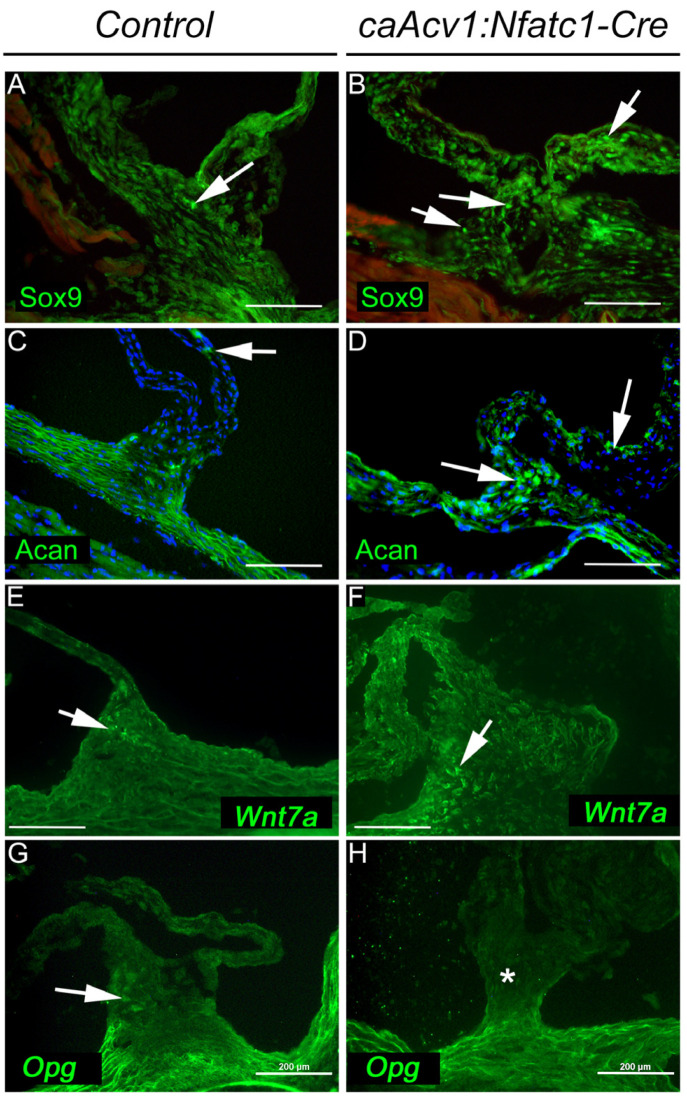
Changes in protein expression and localization in aortic valve tissues between the *caAcvr1:Nfatc1Cre* mutants and controls. Sox9 increased in nuclear localization (arrows in (**A**,**B**)) in the mutants (**B**) compared with the controls (**A**). The mutant aortic valves showed higher Aggrecan (Acan) protein levels (**D**) than the corresponding control tissues (**C**). Arrows in (**C**,**D**) point to positive Acan staining. Wnt7a (white arrows in (**E**,**F**)) showed higher levels in the leaflet–wall junction in the mutants (**F**) than in the controls (**E**). Opg protein was less expressed in the mutants ((**H**), asterisk) than in the controls ((**G**), white arrow). Scale bars, 200 µm; *n* = 6.

In addition, we analyzed the protein localization of Sox9 and Aggrecan (key proteins implicated in the pathogenesis of aortic valve disease), as well as Wnt7a and Opg (which showed altered expression in RNASeq) using immunohistochemistry (Figure 6). Concordant with the increased chondrogenic differentiation in the mutant valve leaflet–wall junctions, we could detect more Sox9 nuclear fluorescence and higher levels of Aggrecan in the mutants than in the controls. Moreover, the overall fluorescence of Wnt7a was higher and Opg was lower in the mutants than in the controls, implying that, at least in the case of these gene products, the observed changes at the RNA level could also be seen at the protein level.

## 4. Discussion

AcvRI-mediated signaling is required for appropriate cardiac and vascular development [28,32,36], while gain-of-function mutations in the *AcvrI* gene result in heterotopic ossification in the rare, but fatal FOP disease [25]. To examine whether AcvRI also plays a role in the formation of calcific aortic valves, we generated mice that display constitutive activation of AcvRI in the endocardium and in non-endocardium-derived cells at the leaflet–wall junction. Our results showed that the mutant *caAcvr1:Nfatc1Cre* mice display thickened sclerotic aortic valve leaflets, chondrogenic changes in the leaflet–wall junction, functional valve defects with variable penetrance, and occasional calcific lesions.

Previous studies have suggested that the morphogenetic signaling pathways involved in cardiac valve development are also involved in the pathogenesis of cardiac valve disease [38]. Mutations in the *Notch1* gene have been shown to result in aortic valve calcification in both humans and animal models [39]. Similarly, BMP signaling has been shown to be instrumental for the appropriate development of cardiac valves [36,40,41]. In addition, mice deficient in the *Acvr1* gene have been shown to display a bicuspid aortic valve phenotype [32], which is a significant risk factor for the development of calcific aortic valve disease in humans. Moreover, a recent study demonstrated overexpression of BMP2 in aortic valve dysfunction and pre-calcific lesions similar to those described in this study [42]. Here, we used the well-characterized *Nfatc1Cre* mouse line, which induces an efficient recombination in the endocardium before and during EndMT [34]. As shown in Figure 1, most of the VICs, as well as cells in the leaflet–wall junction region, which are not derived from the endocardium, show efficient recombination. Despite the efficient recombination, the *caAcvr1:Nfatc1Cre* mutants were viable at birth and did not show any obvious gestational cardiac phenotypes. It has been recently shown that, in intercalated leaflets of semilunar valves, VICs are not derived from the endocardium or neural crest, but from undifferentiated second heart field cells [9]. Therefore, it is likely that the use of Cre drivers that are specific for VICs in intercalated leaflets, e.g., *Tnnt2Cre*, would result in different outcomes. In young *Acvr1:Nfatc1Cre* adults, we could detect both functional aortic valve defects, such as stenosis and insufficiency, and histological defects (mostly sclerotic valve leaflets) with a high frequency. Yet, calcific changes were rarely seen. It is possible that calcific changes would have been more common if we would have studied older animals. However, the *caAcvr1:Nfatc1Cre* mutants had to be euthanized at four months, as the progressive worsening of the limb phenotype [26] made them poorly ambulatory. To date, a few mouse models for calcific aortic valve disease have been described. Among these, a mouse model of premature aging (*Klotho−/−*) and heterozygote *Npr2+/−* and tissue-specific *Sox9* mutants represents models in which genetic modification alone results in aortic valve disease. Most of the other described models, e.g., *Notch1+/−*, *Rbpjk1+/−, Ldlr−/−*, and *Apoe−/−* mutants, only display the phenotype when combined with a special diet. Therefore, we attempted to increase the prevalence of calcific lesions in our *caAcvr1:Nfatc1Cre* mice by feeding the mutants and controls a “pro-calcification” diet [43]. Yet, this diet did not result in observable changes in the valve phenotype. In addition, we tested whether a pro-calcific genetic background could make calcific changes more common. To this end, we crossed our mutant mice with an *Enpp1* background, which has been shown to result in increased vascular calcification [43]. The obtained *caAcvr1:Nfatc1* mutants that were homozygous for *Enpp1 (Enpp1−/−)* died in utero, and thus could not be used to study the progression of aortic valve disease, while *caAcvr2:Nfatc1:Enppi+/−* mice were phenotypically indistinguishable from their *caAcvr1:Nfatc1Cre* littermates.

During aortic valve disease progression, valve leaflet thickening is often the first observed phenotype, followed by inflammation, changes in the valvular extracellular matrix, and fibrosis, ultimately leading to calcification [44]. Our genome-wide transcriptome profiling revealed that the gene sets associated with inflammation and smooth muscle cell contraction were most significantly altered in mutant aortic valve tissues compared with those of the controls. While these changes have been described in both mice and humans with valve disease [45,46], we could not detect alterations in the expression of many genes previously associated with pathogenesis of sclerotic and/or calcific aortic valves. These include the synexpression genes of the Wnt, TGF-beta, and BMP signaling pathways. Similar to primary aortic valve interstitial cell cultures established from human patients suffering from calcific aortic valve disease, *osteoprogeterin* (*Opg*) expression was reduced in our mutants, while elevated *Opg* expression was described in a rabbit model for the stenotic aortic valve [47]. Although the gene encoding a key pro-chondrogenic transcription factor *Sox9* did not show changes in overall expression levels, we could detect increased nuclear Sox9 immunostaining and elevated protein levels of its transcriptional target Aggrecan in mutant tissues, particularly in the leaflet–wall junction, where positive Alcian blue staining and hypertrophic chondrocyte-appearing cells could be seen in the mutants. However, we could not detect consistent changes in expression in the other Sox9 target genes, e.g., *Col2* or *Col10*. In addition, our expression screen showed that the gene sets associated with cGMP signaling were altered in our *caAcvr1:Nfatc1Cre* mutants. cGMP has been shown to mediate C-type natriuretic peptide/NPr2 signaling in valve interstitial cells, and defects in this signaling have been shown promote aortic valve disease in animal models [48]. Therefore, it is possible that constitutive activation of AcvRI contributes to the observed aortic valve phenotype by altering cGMP-mediated signaling processes.

Our expression profiling experiments showed that the BMP synexpression genes were not affected in young adults, suggesting that enhanced BMP signaling via caAcvRI acts either during gestation or during the early postnatal period to cause early sclerotic changes in the valve leaflets in young adult mice. These thickened, stiff leaflets with reduced elasticity would likely result in changes in hemodynamics and shear stress and stronger bending forces on adjacent tissues, particularly at the leaflet–wall junctions, which could then lead to secondary changes in local cellular morphology and matrix deposition.

Gain-of-function mutations in the *Acvr1* gene are causally related to the pathogenesis of FOP, a rare and severe disease characterized by extensive heterotopic ossification (HO) [25]. The most common AcvRI^R206H^ mutation has been intensely studied, and the mechanisms by which it results in FOP are rather well known. In addition to being an atypical BMP type I receptor, wild-type AcvRI functions as a decoy receptor for Activin A [49], which normally signals via AcvRIB to trigger the activation of TGF-ß Smads (Smads2/3) (reviewed in [50]). In contrast, AcvRI^R206H^ responds to Activin by triggering aberrant signaling via BMP Smads (Smads1/5/9) [51]. Interestingly, AcvRI^R206H^ mutations have also been discovered in ~25% of pontine gliomas [52], where AcvRI^R206H^ has been shown to cooperate with mutated Histone H3.1^K27M^ to promote the pathogenesis of the disease [53]. Affected individuals show progressive heterotopic ossification mostly in their muscles and connective tissues (tendons and ligaments), and they usually die in their 30s or 40s [20]. However, aortic valve disease in FOP patients has not been described. Our mouse model provides opportunities to examine the effect of sustained activation of AcvRI-mediated signaling, not only in FOP, but also in other pathologic conditions involving ectopic calcification, e.g., calcific heart valve disease.

## Data Availability

The RNA-Seq data were submitted to the GEO repository under accession number GSE122106.

## Supplementary Materials

The following supporting information can be downloaded at https://www.mdpi.com/article/10.3390/jcdd9080272/s1. Figure S1. Mutant OFT endocardial cushions were not grossly affected at E11.5. Figure S2. Comparison of the aortic valves between adult control and *caAcvr1:Nfatc1Cre* mutant mice.
