# Supplementary material for: Activation of AcvR1-Mediated Signaling Results in Semilunar Valve Defects
Source: J Cardiovasc Dev Dis. 2022 Aug 16;9(8):272. doi: 10.3390/jcdd9080272 (PMC9410128; doi:10.3390/jcdd9080272)
Supplement: Supplementary file 1 [file jcdd-09-00272-s001.zip › jcdd-1806582-supplementary.pdf]

Journal of
*Cardiovascular*
*Development and Disease*

MDPI

**Supplementary Materials**

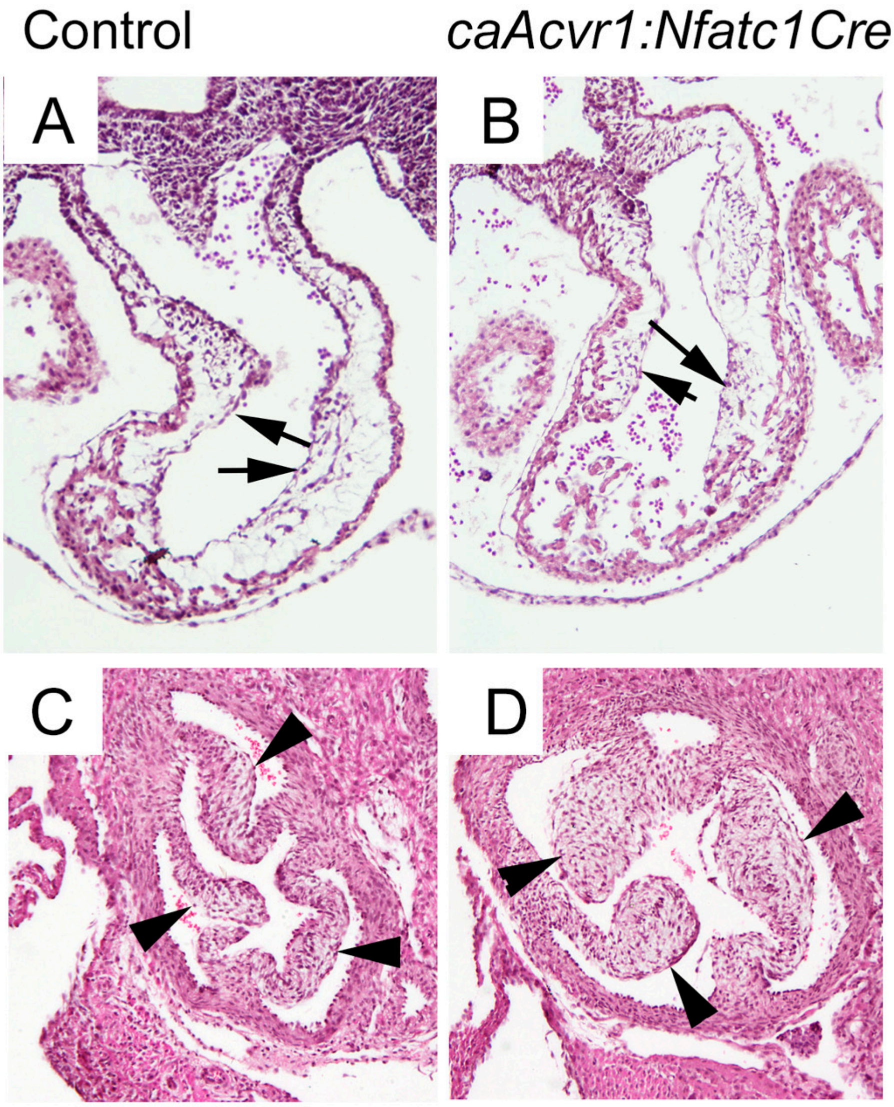


**Figure S1.** Mutant OFT endocardial cushions were not grossly affected at E11.5, while aortic valve leaflets were thickened at P3. The longitudinal section thru the OFT in a control (A) and the caAcvr1:Nfatc1Cre mutant (B) at E11.5. Black arrows point to proximal OFT cushions (A,B). Note that the orientation of samples in A and B is not identical. Cross-sections thru the aortic valves in the control (C) and mutant (D). Black arrowheads point to aortic valve leaflets (C,D). H&E staining.

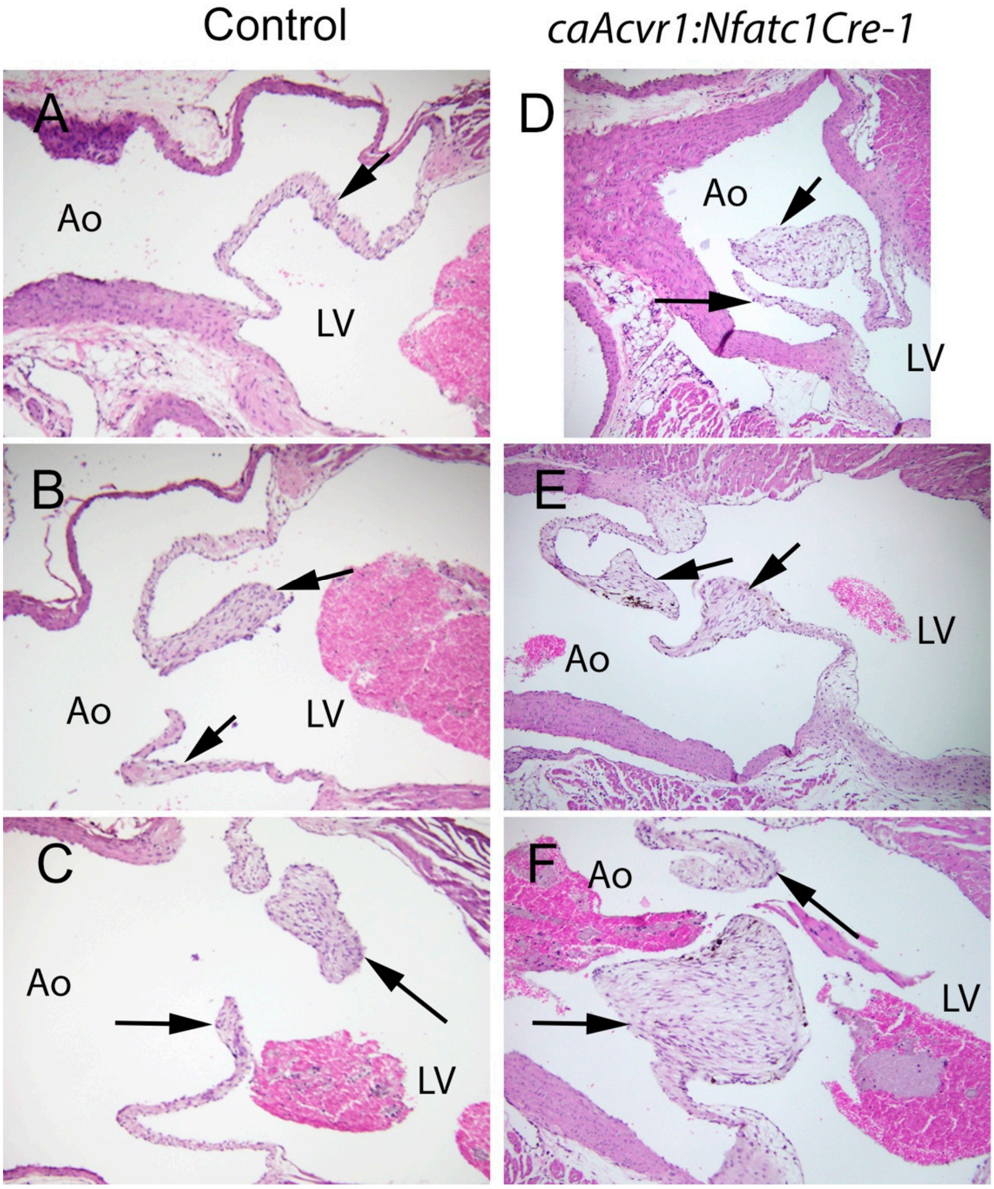


**Figure S2.** Comparison of the aortic valves between adult control and *caAcvr1:Nfatc1Cre* mutant mice. (A–C) Control; (D–F) mutants. Sagittal orientation from right lateral (A,D) to medial (C–F). Black arrows point to aortic valve leaflets (A–F). H&E staining. Ao, aorta; LV, left ventricle. P4 months.
